# Low red blood cell predicts high risk of temporary postoperative complications after carotid body tumor surgical resection

**DOI:** 10.3389/fonc.2022.906048

**Published:** 2022-07-25

**Authors:** Tonglei Han, Shiying Wang, Jiang Zhu, Yudong Sun, Yongfu Xie, Xiaolong Wei, Jian Zhou, Zhiqing Zhao

**Affiliations:** ^1^ Department of Vascular Surgery, Changhai Hospital, The People’s Liberation Army (PLA) Naval Medical University, Shanghai, China; ^2^ Department of Vascular Surgery, Zhongshan Hospital, Fudan University, Shanghai, China; ^3^ Department of General Surgery, Jingling Hospital, Medical School of Nanjing University, Nanjing, China

**Keywords:** carotid body tumor, temporary postoperative complications, red blood cell, hemoglobin, hematocrit, risk prediction model

## Abstract

**Background:**

Carotid body tumor (CBT) is a rare paraganglioma located at the carotid bifurcation. The red blood cell count, hemoglobin, and hematocrit are indexes to be evaluated in blood routine tests. The purpose of this study was to clarify their predictive value for temporary postoperative complications in patients that had undergone CBT surgery.

**Methods:**

This retrospective trial included data from 169 patients received surgical treatment for CBT from October 2008 to September 2018 in this retrospective study. Postoperative follow-up was conducted under the guidance of both vascular surgeon and neurologist. The symptoms existed less than 2 years postoperatively were regarded as temporary injuries. The red blood cell count, hemoglobin, and hematocrit were obtained from the complete blood count results of the participants. Analyses of multilevel multivariable regression and descriptive statistics were conducted.

**Results:**

The baseline data showed no significant difference. Patients were predominantly women (53.8%), with a mean age of 42.6 years. The total incidence of temporary postoperative complications was 22 (13.0%), including transient ischemic attack (8, 4.7%), tongue bias (7, 4.1%), dysphagia (2, 1.2%), hoarseness (4, 1.8%), and eyelid ptosis (1, 2.4%). The univariate and multivariate regression analysis results revealed that the occurrence of temporary postoperative complications was increased with age [odd ratio (OR, 0.09; 95% CI (CI), 0.9–1.0; P = 0.014], length of operation time (OR, 1.0; 95% CI, 1.0–1.0; P = 0.005), Shamblin type II vs. I (OR, 0.1; 95% CI, 0.0–0.5; P = 0.008), red blood cell count postoperative (OR, 0.2; 95% CI, 0.1–0.8; P = 0.026), hemoglobin (OR, 0.9; 95% CI, 0.9–1.0; P = 0.011), and hematocrit (OR, 0.8; 95% CI, 0.7–1.0; P = 0.025). The smooth curve fitting showed that the trend of complications occurrence rate was reduced with the increase of patients’ postoperative red blood cell count, hemoglobin, and hematocrit. Gender, weight, length of operation, Shamblin type, postoperative red blood cell count, hemoglobin, and hematocrit were included in the risk model with AUC = 0.86.

**Conclusion:**

These patients with CBT who received surgical resection with low postoperative red blood cell, hemoglobin, or hematocrit had a high risk of temporary postoperative complications. The risk prediction model established for predicting temporary postoperative complications showed satisfactory prediction effects.

## Introduction

Carotid body tumors (CBTs) are paragangliomas that most commonly originating from the head and neck areas (1, 2). Because of the clinical symptoms and unpredictable malignant potential (systemic metastasis, local compression, erosion of the carotid artery and peripheral nerve tissue, tumor thrombus, etc.) of CBT, surgical management is the preferred treatment for CBT that is better to performed as soon as possible upon diagnosis (3). CBT resection is a relatively risky procedure because of the proximity of the surgical site to several important nerves and blood vessels; its perioperative period complication rate is from 30% to 40% (4, 5).

The two most common complications are cranial nerve injury and intraoperative bleeding (4). Patients with cranial nerve injury will often experience hoarse, dysphagia, and tongue deviation; these deficits that are related to injuries to the vagus nerves, the superior laryngeal nerve, and the hypoglossal nerve (6–9). Whereas some patients have those neurological symptoms for a long time or even permanently, in others, they are just occurring as a temporary phenomenon and most of patients recover before hospital discharge. Another significant complication is intraoperative bleeding, which might affect the normal cranial nerve function or the recovery of the damaged nerves (4). For years, researchers have focused on reducing the incidence of CBT resection complications (10–12). In this study, we found several routine indicators that can predict the occurrence of postoperative adverse events. Taking appropriate measures in a timely manner may reduce the incidence of complications.

## Materials and methods

### Data source

Patients underwent CBT surgical resection in our institution from October 2008 to September 2018 were recruited into this retrospective study and grouped by gender. Computed tomography (CT) was performed for each patient as a necessary routine preoperative examination. All these Shamblin type I, II, and III CBTs were included into this research. Patients were excluded in the following conditions: (1) CBT surgery failed, (2) history of CBT resection, (3) other head and neck radiotherapy or surgery history, (4) carotid artery replacements, and (5) lack of postoperative blood routine examination data.

Their hospital records were reviewed, including preoperative patient profile, intraoperative blood loss, and adverse symptoms after surgery. The postoperative outcome data were collected during 2-year follow-up. These symptoms that still exist 2 years postoperatively were regarded as permanent injuries and not included in the temporary postoperative complications. Temporary postoperative complications after CBT surgery included the following: (1) transient ischemic attack (dizziness, headache, and temporary blurred vision), (2) tongue bias, (3) dysphagia, (4) hoarseness, and (5) eyelid ptosis.

Every included patient’s blood sample was taken at the admission and the second day after CBT surgery. The complete blood routine was assessed by means of an automatic blood counter. Red blood cell count, hemoglobin, and hematocrit were obtained. This trial was approved by the ethics committee and all patients provided written informed consent.

### Procedure

All these patients underwent CBT surgical resection with general anesthesia. The internal jugular vein was identified and the superior thyroid artery was exposed. In general, the vagus nerve accompanying the common carotid artery and the superior laryngeal nerve accompanying the superior thyroid artery can be observed. The hypoglossal nerve can also be seen during some operations. In principle, all nerves should be preserved. These common veins and several cutaneous nerves that would impede the progress of surgery were ligated and cutoff. The CBT was carefully dissected and successfully completely removed according to strict technical standards.

### Analysis and statistics

Continuous variables were reported as means ± SD. Skewed variables were summarized as median and range, depending on distribution of the variables. Group comparisons were analyzed with Student’s t-test or Wilcoxon rank sum test for numeric variables and χ2 or Fisher’s exact test for categorical variables. Generalized linear models with a logit link were used to test the independent and combined effects of these indexes (including red blood cell count, hemoglobin, or hematocrit) and the incidence of temporary postoperative complications with crude or full model adjusted for age, weight, and gender. All analyses were performed using Empower(R) (www.empowerstats.com; X&Y Solutions, Inc., Boston MA) and R (http://www.R-project.org). A P-value of <0.05 was considered to be statistically significant.

## Results

### Patient characteristics

Between October 2008 and September 2018, 169 consecutive patients (78 men and 91 women) entered analysis. Mean patient age was 42.6 ± 12.9 years (range 17 to 75 years). Seventy-five tumors are located on the left side, 52 tumors are located on the right, and 42 patients had bilateral tumors (surgery site, 96; left, 67, right). Length of operation in total was 153.7 ± 71.3 min, a little longer in men (165.9 ± 74.7 min) than in women (143.7 ± 67.2 min), P = 0.049. Intraoperative blood loss shows no significance difference between these two groups, 504.8 ± 633.7 ml in total (**Table 1**). According to the Shamblin classification, tumors were identified: Shamblin type I (n = 22), Shamblin type II (n = 77), Shamblin type III (n = 62), and eight patients’ data lost (**Table 2**).

**Table 1:** Demographic and clinical characteristics of study population.

Patient demographics	Total (n = 169)	Men (n = 78)	Women (n = 91)	P-value
Age, years	42.6 ± 12.9	43.1 ± 12.2	42.2 ± 13.5	0.674
Weight, kg	62.4 ± 11.6	70.1 ± 9.9	55.6 ± 8.3	**<0.001**
Length of operation, min	153.7 ± 71.3	165.9 ± 74.7	143.7 ± 67.2	**0.049**
Blood loss, ml	504.8 ± 633.7	595.2 ± 701.1	426.8 ± 561.7	0.092
Body tumor location				0.867
Left	75 (44.4%)	36 (46.2%)	39 (42.9%)	
Right	52 (30.8%)	24 (30.8%)	28 (30.8%)	
Bilateral	42 (24.9%)	18 (23.1%)	24 (26.4%)	
Shamblin type				0.972
I	22 (13.0%)	10 (13.9%)	12 (13.5%)	
II	77 (45.6%)	35 (48.6%)	42 (47.2%)	
III	62 (36.7%)	27 (37.5%)	35 (39.3%)	
Presenting symptom				
Dysphagia	0 (0.0%)	0 (0.0%)	0 (0.0%)	
Dysphonia	6 (3.6%)	3 (3.8%)	3 (3.3%)	0.847
Family history	4 (2.4%)	1 (1.3%)	3 (3.3%)	0.390
Hypertension	22 (13.0%)	13 (16.7%)	9 (9.9%)	0.192
Diabetes	8 (4.7%)	5 (6.4%)	3 (3.3%)	0.342
Coronary heart disease	4 (2.4%)	4 (5.1%)	0 (0.0%)	0.029
Stroke	4 (2.4%)	3 (3.8%)	1 (1.1%)	0.242
Data lost	8 (4.7%)			

Values are median (interquartile range) or n (%). % are expressed compared to the number of patients. The unpaired t-test was used for analysis between two groups. P values less than 0.05 are bolded in the table.

**Table 2:** Tumor and patient characteristics by Shamblin type.

Patient Demographics	Shamblin Type I (n = 22)	Shamblin Type II (n = 77)	Shamblin Type III (n = 62)	P-Value
Age, years	45.1 ± 14.9	43.6 ± 11.9	40.4 ± 13.7	0.212
Weight, kg	66.3 ± 11.6	63.8 ± 12.0	59.1 ± 10.6	**0.013**
Length of operation, min	103.6 ± 38.6	131.6 ± 60.2	197.5 ± 69.5	**<0.001**
Intraoperative blood loss, ml	260.0 ± 395.1	310.7 ± 358.7	773.4 ± 705.2	**<0.001**
Body tumor location				0.792
Left	12 (54.5%)	20 (48.8%)	21 (42.9%)	
Right	9 (18.2%)	14 (34.1%)	17 (34.7%)	
Bilateral	1 (27.3%)	7 (17.1%)	11 (22.4%)	

Values are expressed as mean ± standard deviation. P-value was calculated comparing different Shamblin type. Group comparisons were analyzed with Wilcoxon rank sum test for numeric variables and Fisher’s exact test for categorical variables. P values less than 0.05 are bolded in the table.

### Analysis of preoperative indicators

Red blood cell count, hemoglobin, and hematocrit before surgery in different gender of patients showed significance difference. Red blood cell count in men was 4.7 ± 0.6 × 10^12^/L but 4.2 ± 0.5 × 10^12^/L in women, P < 0.001. Hemoglobin in men was 144.7 ± 31.1 g/L but 120.3 ± 17.9 g/L in women, P < 0.001. Hematocrit in men was 42.2 ± 3.9% but 36.6 ± 4.6% in women, P < 0.001. Although there was intraoperative bleeding during CBT resection, no significant change could be observed, except red blood cell count, in blood routine at the second day after surgery compared with preoperative. Red blood cell count before surgery in total was 4.5 ± 0.6 × 10^12^/L, compared with 4.3 ± 0.6 × 10^12^/L after surgery, P = 0.022. However, after grouping by gender, there was no significant difference in red blood cell count between preoperative and postoperative (**Table 3**). The statistics of temporary postoperative complications are shown in **Table 4**. No remarkable differences were shown between male and female patients. The total incidence of temporary postoperative complications was 22 (13.0%), including transient ischemic attack (8, 4.7%), tongue bias (7, 4.1%), dysphagia (2, 1.2%), hoarseness (4, 1.8%), and eyelid ptosis (1, 2.4%). Only nine (5.3%) patients with permanent complications could be observed until the end of follow-up, including 1 (0.6%) ipsilateral stroke, 2 (1.2%) tongue bias, and 6 (3.6%) hoarseness.

**Table 3:** Red blood cell count, hemoglobin, and hematocrit before and after surgery in patients with carotid body tumors.

	Before surgery	after Surgery	P-value
Red blood cell count, *10^12^/L			
Total (n = 169)	4.5 ± 0.6	4.3 ± 0.6	**0.022**
Men (n = 78)	4.7 ± 0.6	4.6 ± 0.6	0.091
Women (n = 91)	4.2 ± 0.5	4.0 ± 0.5	0.055
Hemoglobin, g/L			
Total (n = 169)	131.6 ± 27.7	125.8 ± 28.1	0.057
Men (n = 78)	144.7 ± 31.1	137.6 ± 32.4	0.168
Women (n = 91)	120.3 ± 17.9	115.5 ± 18.6	0.084
Hematocrit, %			
Total (n = 169)	39.2 ± 5.1	38.2 ± 5.4	0.072
Men (n = 78)	42.2 ± 3.9	41.1 ± 4.4	0.087
Women (n = 91)	36.6 ± 4.6	35.7 ± 4.9	0.190

Values are expressed as mean ± standard deviation. The unpaired t-test was used for analysis between these indexes before and after surgery. P values less than 0.05 are bolded in the table.

**Table 4:** The incidence of temporary postoperative symptoms.

	Total (n = 169)	Men (n = 78)	Women (n = 91)	P-value
Transient ischemic attack	8 (4.7%)	5 (6.4%)	3 (3.3%)	0.342
Tongue bias	7 (4.1%)	3 (3.8%)	4 (4.4%)	0.858
Dysphagia	2 (1.2%)	1 (1.3%)	1 (1.1%)	0.913
Hoarseness	4 (1.8%)	3 (3.8%)	1 (1.1%)	0.242
Eyelid ptosis	1 (2.4%)	0 (0.0%)	1 (1.1%)	0.353
Total	22 (13.0%)	12 (15.4%)	10 (11.0%)	0.397

N (%). % are expressed compared to the number of patients. The unpaired t-test was used for analysis between men and women.

### Analysis of postoperative indicators

In the univariate regression analysis, a significant association with a higher risk of temporary postoperative complications in age (OR, 0.09; 95% CI, 0.9–1.0; P = 0.008), length of operation time (OR, 1.0; 95% CI, 1.0–1.0; P < 0.001), blood loss (OR, 1.0; 95% CI, 1.0–1.0; P = 0.003), Shamblin type II vs. I (OR, 0.2; 95% CI, 0.0–0.9; P = 0.035), and red blood cell count postoperative (OR, 0.5; 95% CI, 0.2–1.0; P = 0.043). Further multivariate analyses were performed to screen important independent factors. In addition, the following were adjusted in this model: age, gender, weight, length of operation, blood loss, body tumor location, and Shamblin type. Except for blood loss, the occurrence of temporary postoperative complications was still accompanied with an increase of age (OR, 0.09; 95% CI, 0.9–1.0; P = 0.014), length of operation time (OR, 1.0; 95% CI, 1.0–1.0; P = 0.005), Shamblin type II vs. I (OR, 0.1; 95% CI, 0.0–0.5; P = 0.008), and red blood cell count postoperative (OR, 0.2; 95% CI, 0.1–0.8; P = 0.026). Otherwise, hemoglobin (OR, 0.9; 95% CI, 0.9–1.0; P = 0.011) and hematocrit (OR, 0.8; 95% CI, 0.7-1.0; P = 0.025) after adjusted were also found to be independent factors for temporary postoperative complications (**Table 5**).

**Table 5:** Univariate and multivariate regression analysis for temporary postoperative complications.

Variates	Non-Adjusted OR (95% CI), P	Adjusted OR (95% CI), P
Age, years	**0.9 (0.9, 1.0) 0.008**	**0.9 (0.9, 1.0) 0.014**
Length of operation, min	**1.0 (1.0, 1.0) <0.001**	**1.0 (1.0, 1.0) 0.005**
Blood loss, ml	**1.0 (1.0, 1.0) 0.003**	1.0 (1.0, 1.0) 0.637
Shamblin type		
I	1.0	1.0
II	**0.2 (0.0, 0.9) 0.035**	**0.1 (0.0, 0.5) 0.008**
III	1.3 (0.4, 4.5) 0.666	0.4 (0.1, 1.9) 0.231
Red blood cell count * 10^12^/L	**0.5 (0.2, 1.0) 0.043**	**0.2 (0.1, 0.8) 0.026**
Hemoglobin 10 g/L	0.8 (0.6, 1.0) 0.051	**0.9 (0.9, 1.0) 0.011**
Hematocrit %	0.9 (0.9, 1.0) 0.122	**0.8 (0.7, 1.0) 0.025**

Adjust model adjusted for age, sex, weight, length of operation, blood loss, body tumor location, and Shamblin type. Values are expressed as OR (95% CI), P. P values less than 0.05 are bolded in the table.

**Figure 1:**
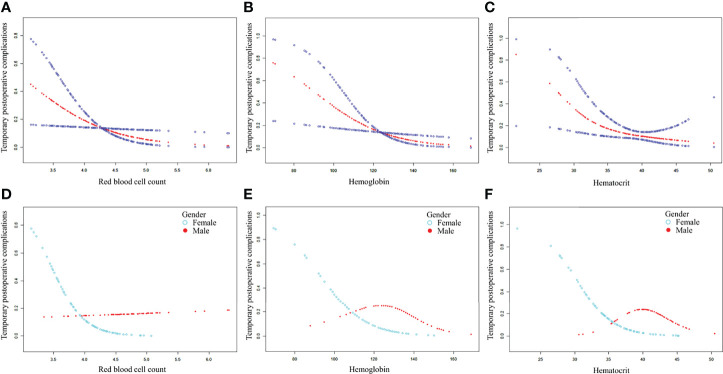
The association between blood routine indexes and temporary postoperative complications. Adjusted for age, gender, weight, length of operation, blood loss, body tumor location, and Shamblin type. **(A–C)** The association between postoperative red blood cell, hemoglobin, or hematocrit and temporary postoperative complications in total patients after CBT surgery. **(D–F)** The association between postoperative red blood cell, hemoglobin, or hematocrit and temporary postoperative complications in male and female patients after CBT surgery. CBT, carotid body tumor.

**Figure 2:**
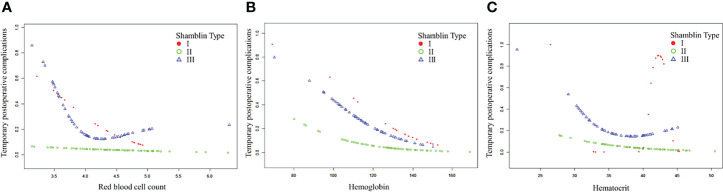
The association between blood routine indexes and temporary postoperative complications in patients with CBT with different Shamblin type. CBT, carotid body tumor. **(A–C)**. The association between postoperative red blood cell, hemoglobin or hematocrit and temporary postoperative complications in CBT patients with different Shamblin type.

After adjusting for possible confounding factors, the smooth curve fitting was performed to explore the relationships (**Figure 1**). An SD increase in red blood cell count postoperative was associated with an 80% reduction in the adjusted risk of temporary postoperative complications (OR, 0.2; 95% CI, 0.1–0.8; P = 0.026), not statistically significant in male patients but still obvious in female patients (OR, 0.0; 95% CI, 0.0–0.5; P = 0.018). An SD increase in hemoglobin was associated with a 10% reduction in the adjusted risk of temporary postoperative complications (OR, 0.9; 95% CI, 0.9–1.0; P = 0.011), still not statistically significant in male patients but remarkable in female patients (OR, 0.9; 95% CI, 0.9–1.0; P = 0.030). The relationship between hematocrit and the incidence of temporary postoperative complications was also statistically significant (OR, 0.8; 95% CI, 0.7–1.0; P = 0.025), still remarkable in female patients (OR, 0.7; 95% CI, 0.5–0.9; P = 0.022). Although there was no significant correlation between these three indicators (red blood cell count, hemoglobin, and hematocrit) and the incidence of temporary postoperative complications in male patients, the trend of complications occurrence rate was still reduced with their increase. In patients with CBT with different Shamblin types, the association between blood routine index and temporary postoperative complications was almost the same (**Figure 2**).

### Risk prediction model and ROC analysis

**Figure 3:**
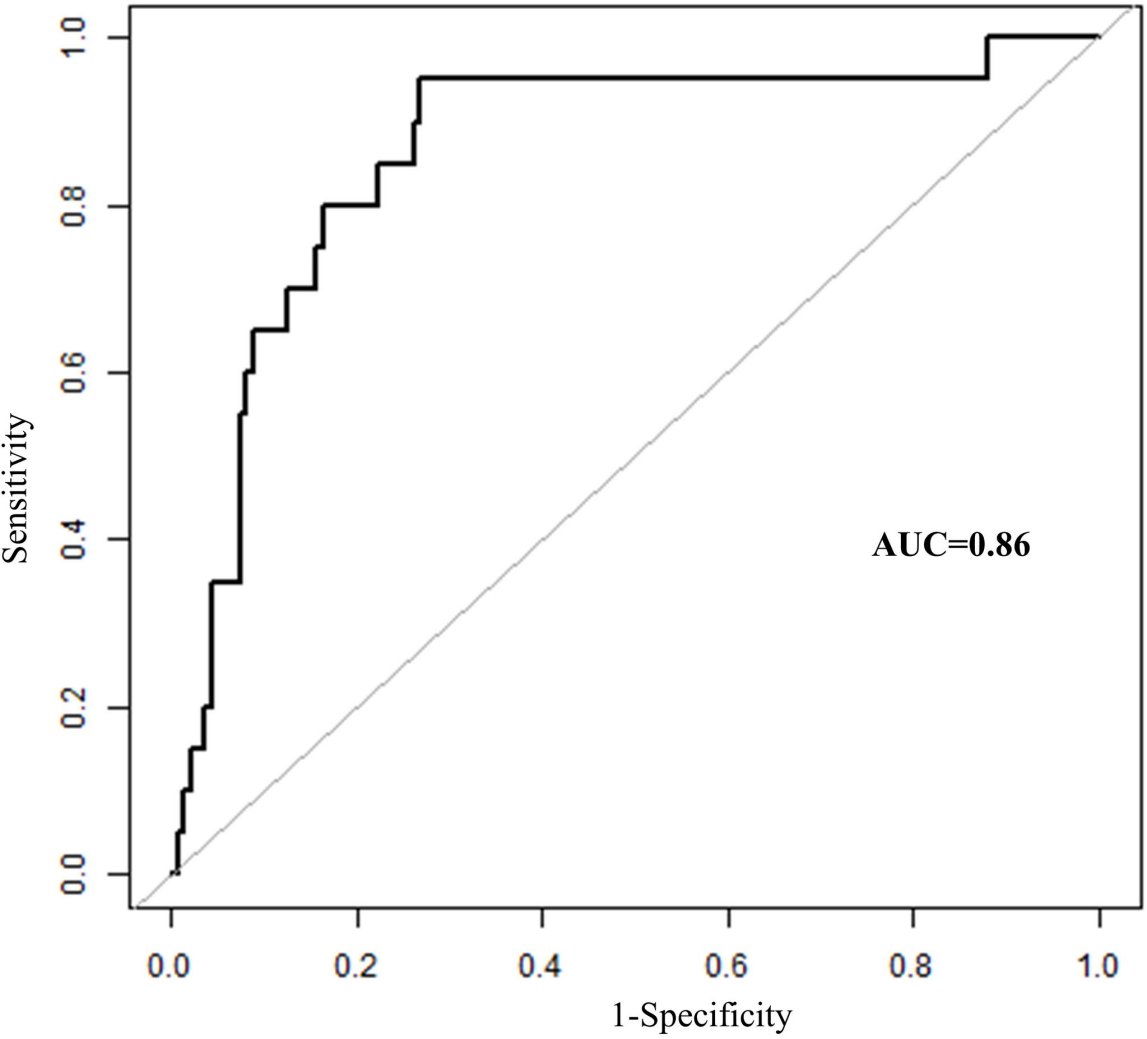
The receiver operating characteristics curves of risk prediction model in predicting temporary postoperative complications after surgical treatment in patients with CBT. CBT, carotid body tumor; AUC, area under ROC curve.

In total, seven parameters were included in the risk model: gender, weight, length of operation, Shamblin type, postoperative red blood cell count, hemoglobin, and hematocrit. With the receiver operating characteristic (ROC) curves, the area under ROC curve (AUC) of this risk prediction model was 0.86 (95% CI = 0.77–0.95) in predicting temporary postoperative complications. On the basis of Youden’s index algorithm in the ROC curve, the optimal cutoff value was 2.18, with sensitivity of 95.0%, specificity of 73.13%, PPV of 34.55%, and NPV of 98.99% (**Figure 3** and **Table 6**).

**Table 6:** The predictive performance of the risk model in predicting temporary postoperative adverse symptoms after CBT surgery treatment.

	Model
AUC	0.86
(95% CI)	(0.77–0.95)
Cutoff value	2.18
Sensitivity (%)	95.00
Specificity (%)	73.13
PPV (%)	34.55
NPV (%)	98.99

AUC, area under ROC curve; CI, confidence interval; PPV, positive predictive value; NPV, negative predictive value. The receiver operating characteristic curve was used for analysis.

## Discussion

CBTs originate mostly from the paraganglia in the head and neck region (13, 14). They always arise at the carotid bifurcation below the mandibular angle and appear as slow-growing and painless masses (11, 15–17). Some patients with CBTs might present hoarseness or dysphagia because of the tumors’ progressive growth, which compress cranial nerves. Partial cranial nerve function can be recovered or compensated after tumor removal, and the symptoms can also disappear. The previous study indicated that CN XII (hypoglossal nerve, 21.9%), CN X (vagus nerve, 20.3%), and recurrent laryngeal nerve (18.8%) were three most commonly injured cranial nerves after surgical resection of CBT (1). Despite being a rare, mostly benign tumor, CBTs have unpredictable malignant potential (18). Thus, early and complete surgical resection is recommended (7, 19). However, the resection of CBTs often adjacent to or surrounded by many carotid arteries and cranial nerves is rather difficult. Moreover, many unpredictable postoperative complications frequently occur (7, 20). Nevertheless, some symptoms disappear over time, including tongue bias, hoarseness, dizziness, headache, and eyelid ptosis.

These temporary postoperative complications in patients with CBTs after surgical resection were caused by nerve injury or cerebral ischemia/reperfusion. The symptoms of some nerve injuries can be surgically recovered, but other symptoms are permanent. Currently, we do not know how these injured cranial nerves are compensated and repaired. No remarkable differences between men and women in the incidence of temporary postoperative complications in patients after CBT surgery was found in our study. The most common temporary symptom in patients after resection of CBTs in our center is transient ischemic attack, followed by tongue bias and hoarseness. These trends indicate that bleeding is the most important factor affecting the occurrence of temporary symptoms.

Abundant evidence has suggested that preoperative embolization in patients with CBTs can reduce intraoperative bleeding. Significant blood loss was associated with the occurrence of severe complications (8, 21). However, relatively few studies have been performed on determining the mechanisms to reduce the incidence of postoperative complications. For example, Bozan et al. have compared the mean platelet volume, red cell distribution width, and neutrophil-to-lymphocyte ratio before and after surgery in patients with CBTs (22). However, the differences in all these indexes were no statistically significant. Moreover, this study did not consider the occurrence of postoperative adverse events and thus did not analyze their association with the parameters investigated.

Intraoperative hemorrhage can decrease the number of red blood cells, as well as the levels of hemoglobin and hematocrit. On the basis of the amount of bleeding in the patient’s CBT surgery, the vascular surgeon decides whether to perform blood transfusion or not. The red blood cell counts of all patients in our study were reduced after CBT surgery, although the differences between male and female patients before and after the surgery were not obvious. Postoperative hemoglobin and postoperative hematocrit were also decreased, but with no statistical significance. Nonetheless, a significant inverse association was established between these three postoperative indexes and the incidence of temporary postoperative complications, especially in female patients. The analysis of the clinical data revealed several potential reasons for this outcome. The red blood cell count in male patients was significantly higher than that in female patients both before and after the surgery. This physiological difference might have retarded the recovery of women’s cranial nerve function. In addition, part of the nerve injury diagnoses was based on patients’ subjective reports, such as transient amaurosis, headache, or dizziness. Women might be more sensitive than men. Smooth curves between postoperative red blood cell count, hemoglobin, or hematocrit and the incidence of temporary postoperative complications stratified by gender are consistent with the smooth curve of all patients. On the basis of the analysis results of the data, we speculated that the incidence of temporary postoperative complications might be reduced with the increase of the postoperative red blood cell, hemoglobin, or hematocrit level of patients with CBT.

A few temporary postoperative complications might develop into permanent injuries. Reducing postoperative complications to minimize or prevent such occurrences could improve patient prognosis. Thus, the early identification of high-risk individuals was very important. This established risk prediction model was easy to implement and would help clinicians in changing subsequent treatment strategies. For these patients with a high risk of temporary postoperative complications, the appropriate amount of iron supplements or blood transfusion was perhaps necessary. Limited by the small sample size and the low incidence of CBT, a prospective research design was difficult to conduct. Our research only revealed several independent factors associated with the occurrence of temporary postoperative complications and provided a risk prediction model. A randomized controlled trial (RCT) or case-control study is required to evaluate this finding.

## Conclusion

The incidence of temporary postoperative complications might be reduced with the increase of the postoperative red blood cell, hemoglobin, or hematocrit counts of patients with CBT, especially in women. The risk prediction model with satisfactory prediction effects could be used for predicting temporary postoperative complications.

## Data availability statement

The datasets used and/or analyzed during the current study are available from the corresponding author on reasonable request.

## Ethics statement

This study was reviewed and approved by the Ethics Committee of Naval Military Medical University. Written approval was waived.

## Author contributions

Conception and design: TH, SW, JZho, and ZZ. Analysis and interpretation: TH, JZhu, and XW. Data collection: TH, YS, and YX. Writing the article: TH, SW, and JZhu. Critical revision of the article: YS, YX, XW, JZho, and ZZ. Statistical analysis: TH and YS. Obtained funding: XW and ZZ. Overall responsibility: ZZ. All authors contributed to the article and approved the submitted version.

## Funding

This study was financed by the Shanghai Clinical Research Youth Project (20184Y0313) and National Natural Science Foundation of China (81770482).

## Conflict of interest

The authors declare that the research was conducted in the absence of any commercial or financial relationships that could be construed as a potential conflict of interest.

## Publisher’s note

All claims expressed in this article are solely those of the authors and do not necessarily represent those of their affiliated organizations, or those of the publisher, the editors and the reviewers. Any product that may be evaluated in this article, or claim that may be made by its manufacturer, is not guaranteed or endorsed by the publisher.
